# Expanding Prevertebral Soft Tissue Swelling Subsequent to a Motor Vehicle Collision

**DOI:** 10.1155/2014/870580

**Published:** 2014-06-26

**Authors:** Matthew F. Ryan, David Meurer, J. Adrian Tyndall

**Affiliations:** Department of Emergency Medicine, University of Florida, 1329 SW 16th Street, P.O. Box 1000186, Gainesville, FL 32610-0186, USA

## Abstract

Cervical acceleration/deceleration or whiplash injuries are a common cause of cervical spine trauma. Cervical acceleration/deceleration can result in vertebral fractures, subluxations, and ligamentous and other soft tissue injuries. Severe injuries are often evidenced by increased prevertebral swelling on lateral X-ray. Assessment of the prevertebral space on lateral cervical spine films is an essential component for identifying potential traumatic neck injuries. We describe a case in which an 84-year-old man on coumadin presented to the emergency department after a low-impact motor vehicle crash. The patient initially complained of neck and shoulder pain which subsequently progressed to hoarseness, dysphagia, and dyspnea. Imaging studies revealed significant prevertebral tissue swelling with anterior compression of his airway that required airway stabilization via awake fiber-optic intubation and reversal of his anticoagulation therapy.

## 1. Introduction

Cervical spine injuries occur in approximately 3.7% of all trauma patients [1]. The injuries are associated with high morbidity and mortality stemming from injuries to the spinal cord and cervical vasculature. A common mechanism of injury is the result of cervical acceleration/deceleration secondary to a blunt impact of such a motor vehicle collision (MVC). This mechanism results in rapid extension of the head followed by forward flexion [2]. A rare consequence of cervical spine injuries is the development of a prevertebral hematoma with associated prevertebral soft tissue swelling. These injuries can lead to rapid airway compromise as a result of significant anterior widening of the retropharyngeal space secondary to edema and the development of a hematoma [3]. Imaging studies can help elucidate the extent of the injury and guide further management. The priority however is to be prepared for establishing a definitive airway if necessary and thus to avoid an airway catastrophe. We describe herein a case of an 84-year-old man who developed large prevertebral tissue swelling after an MVC. Subsequently, the patient required intubation for airway protection. Confounding the case, the patient was on coumadin for atrial fibrillation which needed reversal by using prothrombin complex concentrates (PCC).

## 2. Case Presentation

An 84-year-old man was transported via emergency medical services to the emergency department (ED) following a motor vehicle collision. The patient was the restrained driver and was rear-ended while slowing down to make a turn. There was moderate damage to the rear of the patient's vehicle. The car was an old model vehicle and was not equipped with airbags. The patient was ambulatory at the scene but as a precaution he was transported via emergency medical services to the ED on a backboard and wearing a cervical collar.

On presentation, the patient was in no apparent distress and his vital signs were blood pressure of 157/78, pulse of 82 with a respiratory rate of 18 (unlabored), and pulse oximetry of 97% in room air. The patient initially complained of neck pain, bilateral upper extremity and shoulder pain, and numbness in both hands. A friend who accompanied the patient to the ED noted the patient's voice was raspy sounding. The patient's past medical history was significant for atrial fibrillation for which he took coumadin. He also had an automated implantable cardioverter defibrillator. The patient noted that his implanted defibrillator had not discharged today and he was in his normal state of health prior to the accident.

His physical exam was significant for midline cervical spine tenderness and his anterior neck exam was normal without tracheal deviation, jugular venous distention, or appreciable masses. The patient exhibited no neurological deficits except a complaint of bilateral hand numbness but his motor function was preserved and had full range of motion in all extremities. The patient had multiple abrasions over the anterior portion of both legs and over his right elbow. Laboratory studies revealed the patient had an INR of 3.5.

**Figure 1:**
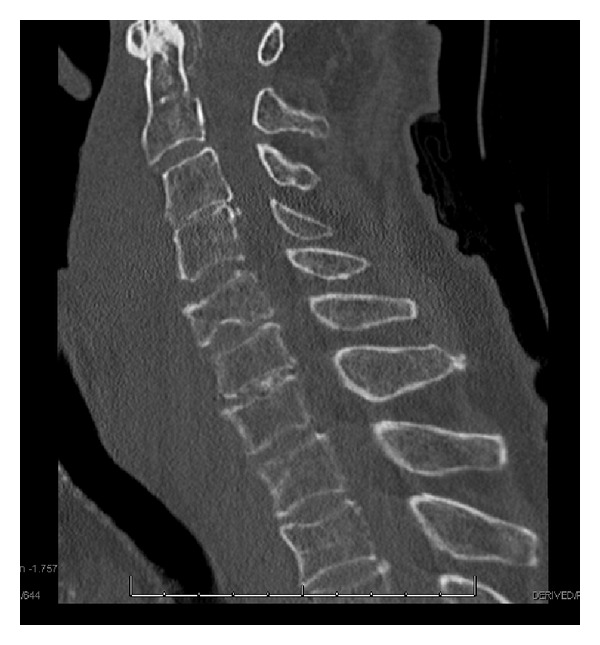
CT soft tissue of the neck without contrast showing prevertebral soft tissue swelling.

**Figure 2:**
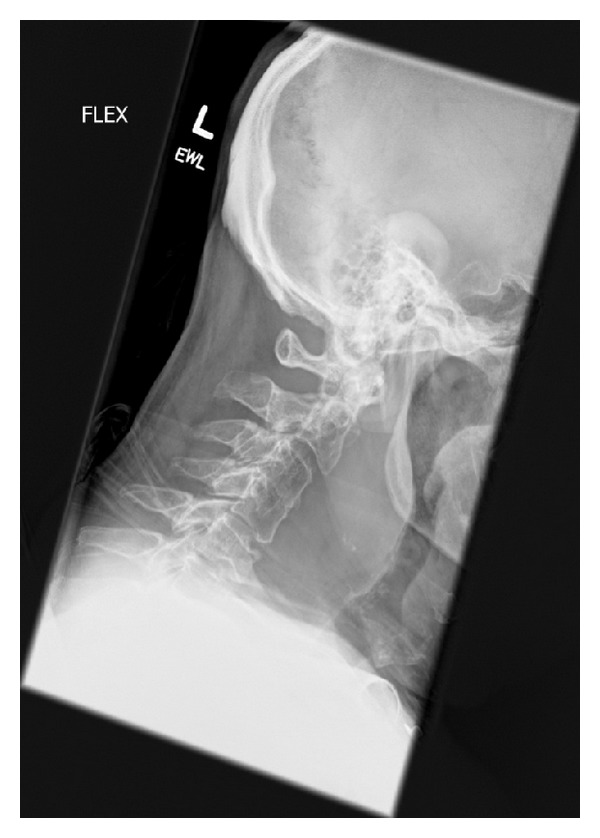
Lateral plain film taking in flexion demonstrating significant prevertebral soft tissue swelling with anterior tracheal displacement.

A chest X-ray and plain film images of the patients shoulders were both normal without evidence of osseous injuries. An extended focused assessment with sonography for trauma was negative for pneumothorax and abdominal, pelvic, and pericardial free fluid. A computed tomography scan (CT) of the patient's cervical spine revealed an anterior C5 compression fracture with significant prevertebral tissue swelling and C2-C3 anterolisthesis. The fracture was causing an acute hematoma in the soft tissues to the right of the trachea and esophagus which extended inferiorly to the level of the right main bronchus (Figure 1). After discussion with neurosurgery and radiology, the decision to further assess the compression fracture was made via lateral plain films (Figure 2); note the patient could not undergo magnetic resonance imaging due to his implanted device.

While in the ED, the patient developed worsening numbness in hands and worsening dysphonia, dysphagia, and difficulty managing his secretions. The decision was made to intubate the patient for potential airway compromise and what was surmised to be an expanding hematoma. Moreover, given the patient's progressively worsening situation, it was decided that the best option would be an awake fiber-optic intubation rather than direct laryngoscopy to minimize the risks of compressing an expanding hematoma or causing its rupture and thus airway compromise.

The patient was sedated with versed 2 mg IV and given 5 mL of 2% viscous lidocaine to drink. Further, 4% lidocaine in saline was atomized into his posterior oropharynx. The fiber-optic scope was passed and after some manipulation and copious suction of secretions which were pooling in the patient's pharynx, the vocal cords were visualized and an 8.0 mm endotracheal tube was passed through the cords without difficulty. Some bleeding was noted during procedure but acute hemorrhage or damage to the cervical hematoma was not evident.

Because the patient's INR was elevated at 3.5 and given his significant airway compromise, the patient was given four-factor prothrombin complex concentrates (PCC) and vitamin K which quickly reversed his hypercoagulable state. Repeat blood work 90 minutes after receiving PCC showed the patient's INR to be 1.3.

Subsequent CT scan of his neck with intravenous contrast noted active contrast extravasation into the prevertebral soft tissues of the neck at the level of C4-5. This was in close proximity to the C4-C5 disc space to the right of midline and was likely stemming from a C5 compression fracture. No arterial feeder was noted and the hematoma was not increasing in size. The patient was later taken to the operating room for incision and drainage of the prevertebral space; however, no hematoma or fluid collection was found and only soft tissue edema was evident.

The patient was admitted to the intensive care unit for continued monitoring and extubated without incident after three days of observation. His oxygen saturation and vital signs remained stable throughout his stay. He was discharged from the hospital after 5 days of observation and has had no subsequent incidence including phonation, dysphagia, or breathing issues since discharge.

## 3. Discussion

Retropharyngeal hematomas are thought to occur rarely [3]. The mechanism of injury can be the result of direct blunt or penetrating neck trauma, disruption of the vasculature of the neck due to aneurysms and dissections, anticoagulation therapy, the presence of tumors or other mass occupying lesions, whiplash (rapid flexion and extension of the neck typically caused from a strong posterior force), and cervical spine fractures [4]. The cause of the prevertebral swelling in this case is thought to be secondary to a whiplash-like injury compounded by the fact that the patient was on a blood thinner and was of advanced age. In general, advanced age predisposes patients to worse outcomes after a trauma as does the use of anticoagulation therapy [1]. The rapid development of an expanding retropharyngeal hematoma with impending airway compromise necessitated immediate intubation in a controlled environment to avoid potentially life-threatening airway obstruction. As the patient was already symptomatic, a coordinated decision was made to perform an awake fiber-optic intubation which provided greater control of the process through direct visualization [5] and avoid any unnecessary airway trauma that may have resulted from direct laryngoscopy. For example, direct laryngoscopy (DL) may lead to rupture of the hematoma and thus increased bleeding, edema, and airway obstruction.

The occurrence of a retropharyngeal hematoma following blunt trauma can be managed conservatively with the use of steroids and observation if the patient is otherwise stable and exhibits no airway or neurological concerns. In cases where a patient's condition precipitously changes, fiber-optic endoscopy has been reported to be the preferred method for intubation. The key is early identification of impending airway compromise through a focused history to obtain mechanism of injury and medical problems and medications and detailed physical exam to corroborate any suspicions of injury [3].

The three potential spaces of the neck are separated by various facial layers from the vertebral bodies and the pharyngeal musculature and larynx moving anteriorly. These spaces are difficult to distinguish radiologically [6]. When examining cervical spine images, the prevertebral soft tissue space is a key point of analysis; the general teaching “6 at 2 and 22 at 6” which denotes the approximate maximum tolerable soft tissue space anterior to the C2 and C6 vertebrae is 6 mm and 22 mm, respectively. A major concern regarding prevertebral swelling identified on radiographs is that these potential spaces can be contiguous with the mediastinum and edema and hemorrhage can spread into the chest as was the case here.

In addition to airway stabilization, the patient's anticoagulation therapy needed to be reversed emergently [7]. The concern here was the rate of expansion of the soft tissue swelling which had already led to airway obstruction. Accordingly, prothrombin complex concentrates (PCC) were administered which lower the patients INR from 3.1 to 1.3 in approximately 90 minutes. Indications for PCCs include rapid reversal of coumadin associated with life-threatening hemorrhage or intracranial bleeding. Because our patient had an expanding hematoma with no obvious source of bleeding and he had an elevated INR, the decision was made to reverse his coumadin. The efficacy of reversal therapy was manifested in the resolution of soft tissue swelling and the absence of drainable hematoma upon incision and drainage.

## 4. Conclusion

Traumatic retropharyngeal hematomas are rare but life-threatening condition. To ensure patient safety, coordinated efforts by skilled personnel are required and should not be delayed. The suspicion of such an injury can be discerned by a focused history and physical exam. Moreover, if a patient has a mechanism for the possibility of existing or delayed retropharyngeal hematomas, close observation for airway obstruction is warranted. It is our recommendations that DL be avoided if possible to avoid disruption of the hematoma and fiber-optic endoscopy be used if available and if operators are technically able to do so. Finally, if anticoagulation therapy contributed to the development of a retropharyngeal hematoma, the use of PCC is also strongly recommended to avert worsening outcomes.
